# ﻿Three new species and one new record of Deimatidae (Echinodermata, Holothuroidea, Synallactida) discovered in the South China Sea and the Mariana fore-arc area using integrative taxonomic methods

**DOI:** 10.3897/zookeys.1195.115913

**Published:** 2024-03-20

**Authors:** Yunlu Xiao, Haibin Zhang

**Affiliations:** 1 Institute of Deep-sea Science and Engineering, Chinese Academy of Sciences, Sanya 572000, China Institute of Deep-sea Science and Engineering, Chinese Academy of Sciences Sanya China; 2 University of Chinese Academy of Sciences, Beijing 100049, China University of Chinese Academy of Sciences Beijing China

**Keywords:** COI, deep-sea, morphology, *Oneirophanta*, phylogeny, sea cucumber, SEM, taxonomy

## Abstract

Deep-sea holothurian specimens were collected during five scientific expeditions (2018–2023) using the submersible vehicle ‘Shenhaiyongshi’. Our examination of specimens of Deimatidae from the South China Sea and the Mariana fore-arc area revealed three new species, which were described as *Oneirophantaidsseica***sp. nov.**, *Oneirophantabrunneannulata***sp. nov.**, and *Oneirophantalucerna***sp. nov.** These species were distinguished from each other and from congeners by the arrangement, and number of ventrolateral tube feet and ossicle types. We also reported *Oneirophantamutabilismutabilis* Théel, 1879 for the first time from the Mariana fore-arc area, and we recorded *Deimavalidumvalidum* for the second time from the South China Sea. The taxonomy of these new species and new records is discussed, and a phylogenetic analysis based on a concatenated dataset of 16S and COI genes was conducted. Additionally, the inter- and intraspecific genetic divergences we calculated among deimatid species. The results support the assignment of these new species to the genus *Oneirophanta* and their separation from congeners. A description of the main morphological characters of *Oneirophanta* species is also provided. The data were collected from geographically diverse areas and suggest that species of Deimatidae were abundant in the Pacific Ocean and occupied a wide range of depths.

## ﻿Introduction

Echinoderms are abundant in Chinese seas, and the South China Sea has more species than the Yellow Sea and the East China Sea (Liao and Xiao 2012). The South China Sea covers ~ 3.5 million km^2^, and it is studded with 200 islands and islets. It is in the center of the Indo-Western Pacific Biogeographic Province, and it is one of the most biodiverse seas in the world. The maximum recorded depth for echinoderms was 5377 m, with considerable habitat and species diversity (Morton and Blackmore 2001; Teh et al. 2019). Deep-sea exploration in China began with the *Jiaolong* manned submersible in 2010, which has been used widely to uncover the deep-sea biodiversity in China seas and the Mariana Trench (Li 2017). Taxonomic research has revealed many new species and new geographical records of animal species in the deep waters of the South China Sea (Li et al. 2019).

Holothurians are the dominant epibenthic invertebrate taxon in many areas of the deep sea, and they account for 90% of that ecosystem’s biomass (Hendler et al. 1995). However, our present knowledge of deep-sea holothurians is still limited due to the difficulty in collecting and depositing good specimens. Even the specimens collected by the submersible carefully and carried from the seabed to the sea surface in seawater-filled containers have arrived aboard the ship in very poor condition (Pawson 1982a). The epidermis and dermis of the whole holothurians begin to slough off, and the entire external body wall is often completely autotomized. The body wall contains a high proportion of water, even the most carefully preserved specimens will shrink greatly, and the shrinkage rate is frequently greater than 90% (Billett 1991). When preserved in absolute ethanol, the holothurians shrink, so all the external characters are easily distorted, which undoubtedly impacts their morphological identification negatively after collection. In recent years, few studies on the order Elasipodida have been conducted in the South China Sea (Li et al. 2018; Xiao et al. 2018, 2023), and studies on other taxa in the South China Sea have also been reported rarely. More morphological information and molecular data should be obtained for a comprehensive taxonomic evaluation of deep-sea holothurians in the South China Sea (Li et al. 2019).

The family Deimatidae formerly belonged in the order Elasipodida Théel, 1882, but was later transferred to the order Aspidochirotida by Smirnov (2012). Miller et al. (2017) subdivided paraphyletic ordo Aspidochirotida into three separate orders and placed family Deimatidae to the order Synallactida Miller, Kerr, Paulay, Reich, Wilson, Carvajal & Rouse, 2017. The order Synallactida includes the families Deimatidae Théel, 1882, Stichopodidae Haeckel, 1896, and Synallactidae Ludwig, 1894. Deimatidae is the smallest family in the order, and it contains 13 accepted species within three genera (*Oneirophanta* Théel, 1879, *Orphnurgus* Théel, 1879, and *Deima* Théel, 1879) (WoRMS 2023). Only two of the recognized species in the family Deimatidae, *Orphnurgusprotectus* (Sluiter 1901b) and *Deimavalidum* Théel, 1879, have been recorded from the South China Sea (Liao 1997). The genus *Oneirophanta* was established with *Oneirophantamutabilis* Théel, 1879 as the type species (Théel 1879). Currently, there are only three species of *Oneirophanta*, and none of them have been found in China. Eleven deimatid specimens were collected from the South China Sea and the Mariana fore-arc area from 2018 to 2023. Morphological observations of these specimens suggested that they represented three new species and one new record from the Mariana fore-arc area, and they all belonged to the genus *Oneirophanta*.

In addition, we present a morphological description of *Deimavalidumvalidum* Théel, 1879, which was recorded for the second time in the South China Sea; the present specimens show some variations compared with specimens that were recorded previously. Our study provides comprehensive a description of morphological characters, an assessment of intraspecific divergence between the new species and all other known species, and more molecular details that may be useful for further studies of the phylogeny and diversity of the family Deimatidae.

## ﻿Materials and methods

### ﻿Sampling and preservation

**Figure 1.**
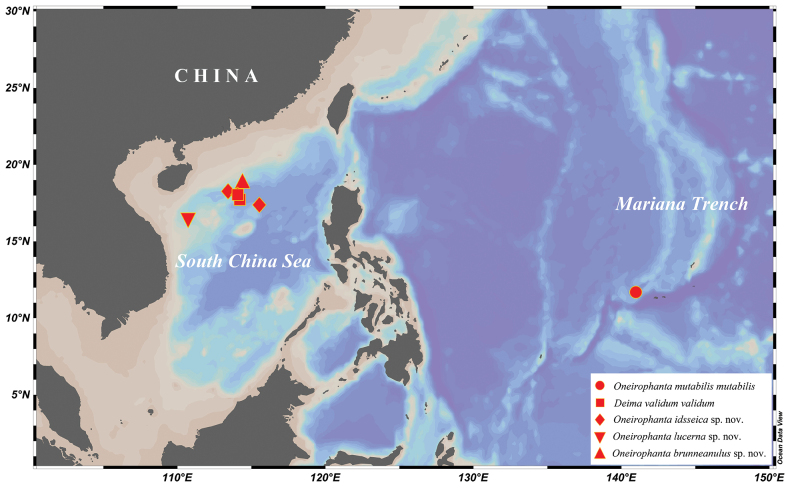
Sampling sites for examined species of Deimatidae in the South China Sea and the Mariana fore-arc area.

Specimens were collected from the South China Sea and the Mariana fore-arc area (Fig. 1) using the manned submersible vehicle ‘Shenhaiyongshi’ from 2018 to 2023, at depths of 1340–3806 m. Samples were frozen or preserved in absolute ethanol, and then stored at the Institute of Deep-sea Sciences and Engineering (**IDSSE**), Chinese Academy of Sciences (**CAS**), Sanya, China.

### ﻿Morphological observations

The specimens of each species were identified using a variety of original descriptions and literature (Théel 1879; Koehler and Vaney 1905; Hansen 1967, 1975; Pawson 2002). External morphological features were examined under a dissecting stereomicroscope (OLYMPUS SZX7), and identification was based on in situ images or pictures photographed in our lab using a Canon EOS 6DII camera. To prepare the deposits, small pieces of body tissue (dorsal and ventral body wall, tentacles, papillae, and tube feet) were digested in a 15% sodium hypochlorite solution. The deposits were then washed three times in distilled water and dried in absolute ethanol before examination with a scanning electron microscope (Phenom ProX).

### ﻿DNA extraction, PCR amplification, and DNA sequencing

Total genomic DNA was extracted from small pieces of 20–30 mg holothurian muscle tissue using a TIANamp Marine Animals DNA Kit (TianGen, Beijing), according to the manufacturer’s instructions. Mitochondrial cytochrome c oxidase I (COI) and 16S rRNA were generated for various specimens using the primers and methods outlined in Miller et al. (2017). The PCR amplification program was as follows: initial denaturation at 98 °C for 3 min, followed by 40 cycles at 98 °C for 10 s, 52 °C for 10s, 72 °C for 10s, and a final extension at 72 °C for 5 min. Total reaction volume was 50 μL: 25 μL Premix Taq with 1.25U Taq, 0.4 mM of each dNTP, 4 mMMg2+ (Ex Taq version, Takara, Dalian, China), 0.5 μM each of the primers and ~ 100 ng template DNA. The sequence chromatograms were then checked using CHROMAS 2.23 (Technelysium Pty Ltd.). The forward and reverse sequences were assembled using CONTIG EXPRESS, which is a component of Vector NTI Suite 6.0 (Life Technologies, Carlsbad, California).

### ﻿Phylogenetic analyses

Two partial sequences (COI and 16S) were obtained from specimens and were deposited in GenBank (Table 1), and some relevant sequences (from all available deimatid species) that were downloaded from BOLD (https://www.boldsystems.org/) and NCBI (https://www.ncbi.nlm.nih.gov/) databases were used for phylogenetic analyses. *Apostichopuscalifornicus* and *A.parvimensis* in the family Stichopodidae (order Synallactida) were used to root the tree.

**Table 1.** List of GenBank accession numbers for all specimens used in this study.

Species	GenBank accession number		Reference
	16S	COI	
**Deimatidae**			
*Orphnurgusglaber*	KX856746	KX874361	Miller et al. 2017
*Deimavalidum*	KX856744	KX874364	Miller et al. 2017
*Deimavalidumvalidum* SY155-HS01	N/A	OR413734	this study
*Deimavalidumvalidum* SY84-HS02	OR658899	OR413743	this study
*Oneirophantasetigera*	KX856745	KX874363	Miller et al. 2017
*Oneirophanta* stet. CCZ_100	N/A	ON400706	Bribiesca-Contreras et al. 2022
Oneirophantacf.mutabilis	ON406619	ON400724	Bribiesca-Contreras et al. 2022
*Oneirophantaidsseica* sp nov. SY86-HS01	OR658900	OR413744	this study
*Oneirophantaidsseica* sp. nov. SY84-HS01	OR658898	OR413742	this study
*Oneirophantaidsseica* sp. nov. SY283-HS01	OR658902	OR413737	this study
*Oneirophantabrunneannulata* sp. nov. SY157-HS01	OR658901	OR413733	this study
*Oneirophantamutabilis* SY310-HS01	OR658897	OR413735	this study
*Oneirophantalucerna* sp. nov. SY529-HS02	OR658906	OR413738	this study
*Oneirophantalucerna* sp. nov. SY530-HS01	OR658903	OR413739	this study
*Oneirophantalucerna* sp. nov. SY530-HS02	OR658904	OR413740	this study
*Oneirophantalucerna* sp. nov. SY530-HS03	OR658905	OR413741	this study
**Outgroups**			
*Apostichopuscalifornicus*	DQ777096	HM542319	Miller et al. 2017
*Apostichopusparvimensis*	KX856750	KX874373	Miller et al. 2017

Sequence alignments were generated using MAFFT7 (Katoh and Standley 2013) with default parameters. Gblocks (Talavera and Castresana 2007) were used to remove batches of fragments from two alignments that were aligned ambiguously. The best partitioning scheme and evolutionary models for two pre-defined partitions were selected using PartitionFinder2 (Lanfear et al. 2017), with all algorithms and AICc criteria. Maximum likelihood phylogenies (ML) were inferred using the Shimodaira-Hasegawa-like approximation likelihood-ratio test (Gascuel 2010) and IQ-TREE (Lam-Tung et al. 2015) models with 20,000 ultrafast bootstraps (Minh et al. 2013). Bayesian Inference phylogenies (BI) were inferred using MrBayes 3.2.6 (Ronquist et al. 2012) under the partition model (two parallel runs, 5,000,000 generations). The initial 25% of sampled data were discarded as burn-in, and the remaining trees were summarized in a 50% majority rule consensus tree. The results were visualized using FigTree v. 1.4.4. The Kimura two-parameter (K2P) genetic distances of COI among deimatid species were calculated using model MEGA X (Kumar et al. 2018).

## ﻿Results

### ﻿Taxonomy

**Order Synallactida Miller, Kerr, Paulay, Reich, Wilson, Carvajal & Rouse, 2017**

**Family Deimatidae Théel, 1882**

**Genus Oneirophanta Théel, 1879**

#### ﻿ Oneirophanta idsseica sp. nov.

Taxon classificationAnimaliaSynallactidaDeimatidae

69E3056A-004F-5C0A-B7F5-FE127727A7A6

https://zoobank.org/50ADB642-A7E7-476A-AE6C-B45C2803F272

Figs 2 , 3

Oneirophanta stet. CCZ_100, Bribiesca-Contreras et al. 2022: 64–65, fig. 40.

##### Type material.

***Holotype*.**IDSSE-2018-0612-HS01, collected from the Xisha Trough of the South China Sea, station SY86-HS01 (18°16.11'N, 113°25.32'E), depth 2985 m, 12 Jun. 2018, preserved in absolute alcohol. ***Paratypes*.** Two specimens. IDSSE-2018-0531-HS01, collected from the Xisha Trough of the South China Sea, station SY84-HS01 (18°2.70'N, 114°3.51'E), depth 3156 m, 31 May 2018, preserved at -80 °C. IDSSE-2020-0917-HS01, collected from the northern slope of the South China Sea, station SY283-HS01 (17°23.20'N, 115°32.32'E), depth 3806 m, 17 Sep. 2020, preserved in absolute alcohol.

##### Type locality.

In the Xisha Trough, which is located in the northern slope of the South China Sea, depth 2985 m.

##### Diagnosis.

Body elongated and cylindrical, color yellowish-white. Tentacles 15. Ventrolateral tube feet up to 40–50 pairs, in alternating two or three rows. Dorsal papillae 18–20 on each side, in single rows. Ventrolateral papillae 9–12 on each side, in single rows. Midventral tube feet two and rudimentary. Dorsal deposits irregular perforated plates and varying types of crosses. Perforated plates and crosses with open ramifications ventrally. Papillae deposits slender and sturdy rods with spatulated ends, and crosses with open ramifications. Spatulated rods and irregular deposits in tube feet. Sturdy spatulated rods in tentacles.

##### Description of holotype.

**Figure 2.**
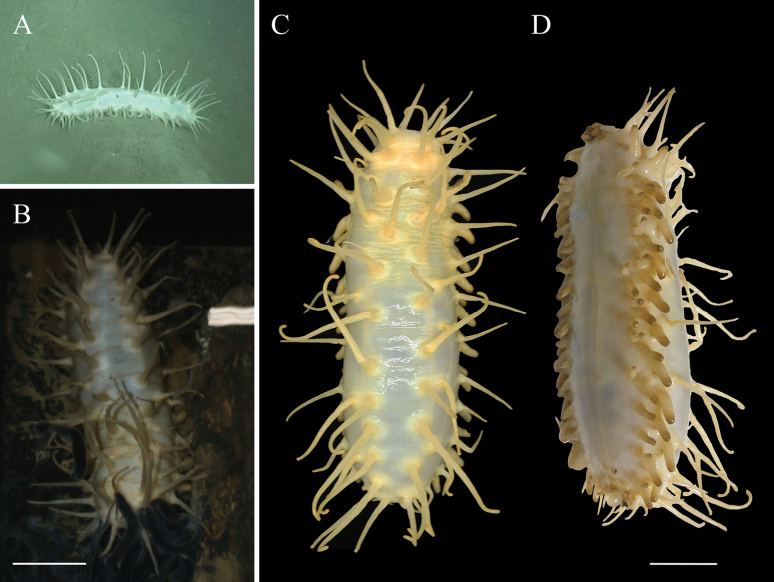
*Oneirophantaidsseica* sp. nov. (Holotype: IDSSE-2018-0612-HS01) **A** in situ image **B** holotype in live **C** dorsal view **D** ventrolateral view. Scale bars: 5 cm.

***External morphology*.** Body elongated and cylindrical, ventrum flattened. 29 cm long and 9 cm wide before fixation (Fig. 2A, B). Color in vivo yellowish-white, tentacles, papillae, and tube feet often darker (Fig. 2C, D). Mouth anteroventral, anus posteroventral. Tentacle discs with rounded knobs at the edges, and the tentacles partially retracted into the mouth, making the number of tentacles difficult to calculate. Ventrolateral tube feet 40 pairs, arranged in alternating two or three rows on each ventrolateral ambulacrum. Midventral tube feet two and rudimentary, scattered along the mid-ventral ambulacrum, one positioned in the front third of the body, the other is positioned in the back third of the body, the anus surrounded by few small tube feet. Dorsal papillae roughly 18–20 on each body side, evenly distributed, measuring 4–10 cm in length, and placed in single rows along the dorsal radii. Ventrolateral papillae ~ 9–12 on each side, generally shorter than dorsal papillae, measuring 2.5–7 cm in length, arranged in single rows along the ventrolateral radii. All papillae slender and tapering from base to end.

**Figure 3.**
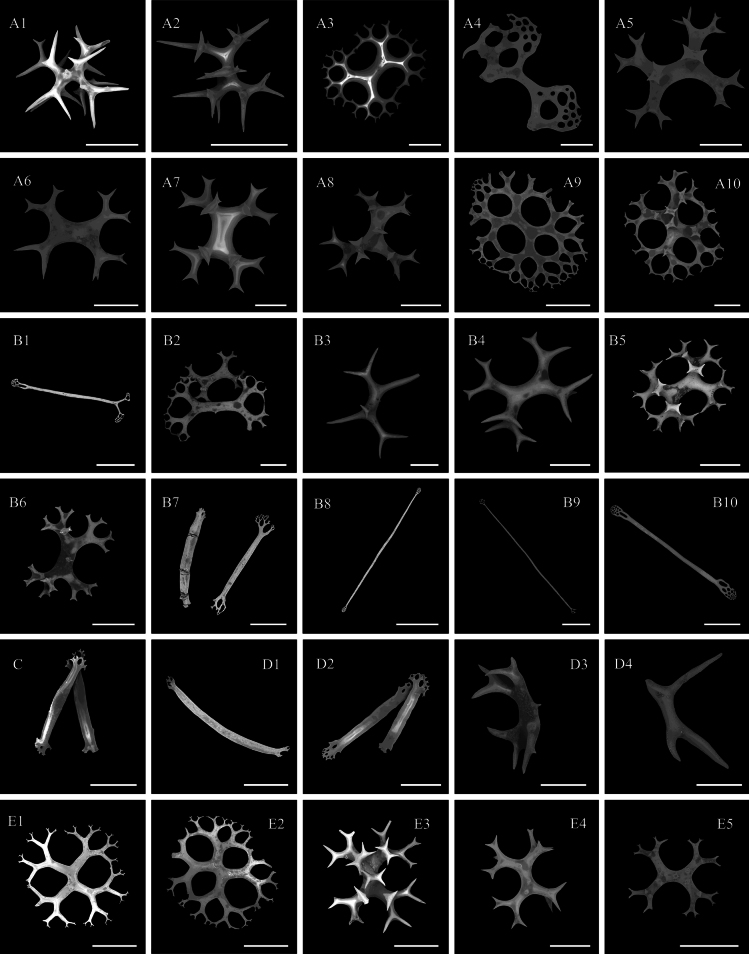
SEM images of different tissues from *Oneirophantaidsseica* sp. nov. (Holotype: IDSSE-2018-0612-HS01) **A1–A10** dorsal body wall **B1–B10** papillae **C** tentacles **D1–D4** tube feet **E1–E5** ventral body wall. Scale bars: 50 μm (**A1, A2, A7, B3, B4, D3, D4, E4**); 100 μm (**A3–A6, A8, A10, B2, B5, B6, E3, E5**); 300 μm (**A9, B1, B7–B10, C, D1, D2, E1, E2**).

***Ossicle morphology*.** Dorsal deposits contain (1) perforated plates with open ramifications (Fig. 3A3, A9, A10), 0.3–0.8 mm in diameter, bearing 0–5 processes on the surface, central holes smaller towards the edge. (2) crosses two types, the first with dichotomously ramified ends, one side bearing 2–5 processes and the other side smooth and without spines (Fig. 3A5–A8); the second irregular, with arms slender than the first type and numerous bifurcated spines throughout the length (Fig. 3A1, A2). (3) spatulated rods with enlarged ends (Fig. 3A4). Papillae deposits contain (1) extremely slender or sturdy spatulated rods, 0.8–1.6 mm long (Fig. 3B1, B7–B10); (2) spatulated crosses with 2–3 processes on the surface (Fig. 3B2, B5, B6); (3) crosses with open ramifications resembling dorsal deposits (Fig. 3B3, B4). Deposits in tentacles only spatulated rods (Fig. 3C), more robust than spatulated rods in papillae. Tube feet deposits mainly robust spatulated rods (Fig. 3D1, D2), 0.6–0.8 mm long, and irregular deposits, possibly in the developmental stage of perforated plates (Fig. 3D3, D4). Deposits in the ventrum same as dorsal (Fig. 3E1–E5), except for large spatulated rods with enlarged ends (Fig. 3A4).

##### Etymology.

Consists of IDSSE and the Latin suffix *icus* (belonging to), to honor IDSSE’s contributions and efforts to the field of deep-sea exploration.

##### Distribution.

A seamount in APEI 4, Clarion Clipperton Zone; Xisha Trough, the northern slope of the South China Sea, at depths of 2985–3806 m.

##### Remarks.

*Oneirophantaidsseica* sp. nov. is characterized by the arrangement of ventrolateral tube feet in two or three rows that number up to 40–50 pairs with distinctive cross-types in dorsal deposits. *O.idsseica* sp. nov. is distinct from *Oneirophantasetigera* (Ludwig 1893) due to the presence of small, perforated plates and crosses with open ramifications that are usually bifurcated. *O.idsseica* sp. nov. differs from *Oneirophantaconservata* Koehler & Vaney, 1905 and *Oneirophantamutabilis* Théel, 1879 by the arrangement and high number of ventrolateral tube feet, and the absence of large, perforated plates on dorsum.

The phylogenetic trees showed that *O.idsseica* sp. nov., together with an unnamed species (*Oneirophanta* stet. CCZ_100, see below), formed a sister group that included Oneirophantacf.mutabilis and *O.mutabilis*. From a morphological point of view, *O.idsseica* sp. nov. mostly resembled *O.* stet. CCZ_100 with ventrolateral tube feet arranged in two or three rows, two rudimentary midventral tube feet, spatulated crosses and small, irregular perforated plates on dorsum, and crosses with open ramifications in different stages of development on the ventrum. From a molecular point of view, the COI pairwise distance between *O.idsseica* sp. nov. and *O.* stet. CCZ_100 was 0.6% (Suppl. material 1). Compared with the description of *O.* stet. CCZ_100, our study provides more details of ossicle morphology of tentacles, tube feet, and papillae.

#### ﻿ Oneirophanta brunneannulata sp. nov.

Taxon classificationAnimaliaSynallactidaDeimatidae

041AC48A-C762-5311-B3BA-6AE14B703662

https://zoobank.org/97066926-74E7-4525-A25D-0F047F525BCC

Figs 4 , 5

##### Type material.

***Holotype*.**IDSSE-2018-0612-HS01, collected from the continental slope of the South China Sea, station SY157-HS01 (18°51.18'N, 114°24'E), depth 1340 m, 1 Jul. 2019, preserved in -80 °C

##### Type locality.

On the continental slope of the South China Sea, depth 1340 m.

##### Diagnosis.

Body elongated, color reddish brown, with darker tentacles and tube feet. Mouth and anus ventral. Tentacle 20. Ventrolateral tube feet ~ 37 pairs, each tube foot end with a brown ring, arranged in alternating three rows, bilateral symmetry. Dorsal papillae 23–26 on each body side, arranged in single rows. Ventrolateral papillae 9–11 on each body side. Midventral tube feet two and rudimentary. Deposits perforated plates, rods of varying shapes and few spatulated crosses.

##### Description of holotype.

**Figure 4.**
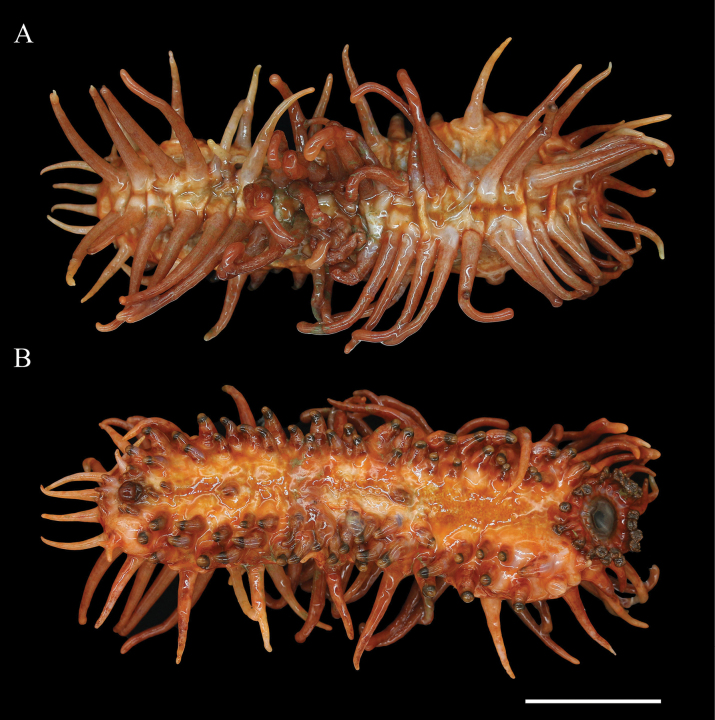
*Oneirophantabrunneannulata* sp. nov. (Holotype: IDSSE-2018-0612-HS01) **A** dorsal view **B** ventral view. Scale bar: 6 cm.

***External morphology*.** Body elongated, dorsum convex, ventrum flattened. 20 cm long, and 5 cm wide before fixation (Fig. 4A, B). Skin reddish brown, with darker coloration on tentacles and tube feet, a brown ring at the end of each tube foot. Tentacle 20, never with ramified processes, circum-oral papillae absent. Ventrolateral tube feet ~ 37 pairs, placed in alternating three rows, bilateral symmetry. Midventral tube feet two and rudimentary, one placed on half the body, the other placed on a rear quarter of the body, and several small tube feet surrounding anus. Dorsal papillae 23–26 on each body side, arranged in single rows along dorsal radii, measuring 5–6 cm. Ventrolateral papillae 9–11 on each side, arranged in single rows, comparatively shorter than dorsal papillae, measuring 2.3–4.6 cm.

**Figure 5.**
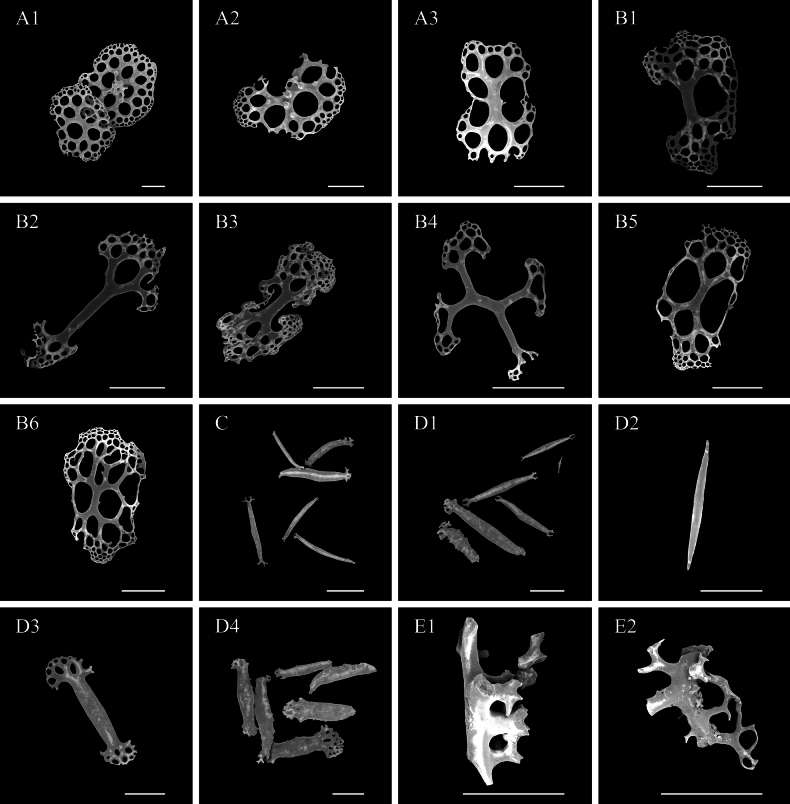
SEM images of *Oneirophantabrunneannulata* sp. nov. (Holotype: IDSSE-2018-0612-HS01) **A1–A3** dorsal body wall **B1–B6** papillae **C** tentacles **D1–D4** tube feet **E1–E2** ventral body wall. Scale bars: 300 μm.

***Ossicle morphology*.** Dorsal deposits only robust perforated plates present (Fig. 5A1–A3), 0.6–1.1 mm in diameter, with central holes tapering from the center to the end, some perforated plates with irregular central apophysis (Fig. 5A1). Papillae contain (1) robust spatulated rods (Fig. 5B1–B3), 0.8–1 mm in length and partially connected at the ends (Fig. 5B1); (2) spatulated crosses (Fig. 5B4); (3) Perforated plates with 2–4 large central holes, rather slender than dorsal, measuring an average 0.9 mm in diameter (Fig. 5B5, B6). Tentacle deposits slender and sturdy rods with open ramifications (Fig. 5C), 0.4–0.6 mm in length. Tube feet with varying types of rods: (1) slender rods with open ramifications, 0.5–0.7 mm long (Fig. 5D1); (2) smooth spindle-shaped rods without spines, 0.7 mm in length (Fig. 5D2); (3) sturdy spatulated rods (Fig. 5D1, D3, D4), with rudimentary or enlarged ends, 0.5–0.9 mm in length. Numerous amorphous shaped, irregular broken deposits on ventrum (Fig. 5E1, E2).

##### Etymology.

The specific epithet *brunneannulata* in Latin means brown rings. It is here used as a noun in apposition and refers to the distinctive brown rings around the tube feet.

##### Distribution.

Only in the type locality.

##### Remarks.

*Oneirophantabrunneannulata* sp. nov. differs from other species in the genus in possessing brown rings at the end of tube feet that are arranged in three rows along ventrolateral radii. *O.brunneannulata* sp. nov. is relatively similar to *O.mutabilis* in possession of plates and spatulated rods, but there are differences: (1) different diameters of perforated plates, 0.6–1.1 mm in *O.brunneannulata* sp. nov., but 2–3 mm in *O.mutabilis*, and some perforated plates in *O.brunneannulata* sp. nov. possess a central apophysis. (2) different types of deposits in tube feet, *O.brunneannulata* sp. nov. has various forms of spatulated rods and a few spindle-shaped rods, but lacks perforated plates, and there are perforated plates in addition to sturdy and spatulated rods in *O.mutabilis*. *O.brunneannulata* sp. nov. differs from *O.setigera* in having perforated plates on the dorsum and the ventrum, lacked spatulated crosses, and had a large number of tube feet that were arranged in three rows. Larger central perforations on perforated plates were in papillae, and the presence of spatulated rods and papillae were arranged in single rows along the dorsal radius (double rows along dorsal radius in *O.conservata*) distinguished *O.brunneannulata* sp. nov. from *O.conservata* (Table 2).

**Table 2.** Main morphological characters in species of *Oneirophanta* Théel, 1879.

Characteristics	*O.idsseica* sp. nov.	*O.brunneannulata* sp. nov.	*O.lucerna* sp. nov.	*O.conservata*	*O.setigera*	*O.mutabilismutabilis*	*O.mutabilisaffinis*
Tentacle number	15	20	19 or 20	Only eight founded	15–20	18–20	18–20
Ventrolateral tube feet number and arrangement	40–50 pairs, in alternating two or three rows	~ 37 pairs, in alternating three rows	11–14 on each side, in single rows	33–34 on each side, in two rows	16–30 on each side, in alter-nating double rows	8–28 (36), in alterna-ting double rows	15–20 (44), –
Dorsal papillae number and arrangement	18–20 on each side, in single rows	23–26 on each body side, in single rows	15–27 on each body side, in regular single rows	30, in double rows	12–32, in irregular double rows	4–19, in single rows	5–35, in single or double rows
Ventrolateral papillae number and arrangement	9–12 on each side, in single rows	9–11, in single rows	7–10, in single rows	13, in two rows	9–17, –	4–17, –	5–11, –
Midventral tube feet number and arrangement	Two and rudimentary, one positioned in the front third of the body, the other positioned in the back third of the body	Two, rudimentary, one placed on half the body, the other placed on a rear quarter of the body	Two and rudimentary, one placed on half the body, the other on a rear quarter of the body	12, placed through-out the entire length of this radius, sometimes in pairs	0–6, in front of the anus	0–4, pre-anal	3–9, usually one pair placed pre-anal
Dorsal Deposit	Perforated plates with open ramifications and crosses with dichotomously ramified ends or irregular, and spatulated rods	Perforated plates, 0.6–1.1 mm in diameter, some with irregular central apophysis	Spatulated crosses, spinous rods with branched spines, spatulated rods up to 1 mm	Perforated plates, some in the developmental stage, rods with slightly thorny surfaces, pointed or bifid ends occasionally	Spatulated crosses, 1.1–3 mm	Perforated plates, 2–3 mm in diameter, bearing several small, vertical spines, with a rather slender mesh-work	Robust and rather small, vertical spines, often irregularly shaped due to elongation of the primary rod
Ventral	Perforated plates and crosses with open ramifications	Numerous irregular broken deposits, amorphous shaped	Spinous rods and spatulated crosses with arms twice divided	–	Spatulated crosses, 0.2–2.3 mm	Vary more, less well-developed, and less irregular than dorsal ones	–
Papillae	Slender or sturdy rods, crosses with open ramifications, some bearing 2–3 processes	Perforated plates with 2–4 large central holes, robust spatulated rods and few spatulated crosses	Spinous rods and spatulated rods		Spatulated rods	Perforated plates only	Perforated plates, small, sturdy and spatulated rods
Tube feet	Mainly robust spatulated rods and irregular deposits	Smooth spindle-shaped rods, slender rods with dichotomously ramified ends, sturdy spatulated rods with rudimentary or enlarged ends	Spinous rods of two types, one irregularly shaped, the other with few regularly distri-buted spines, sturdy spatulated rods with perforated extremities that occasionally bifurcated		–	Perforated plates	Sturdy and spatulated rods
Tentacle	Sturdy spatulated rods	Slender and sturdy rods with open ramifications			–	Irregularly placed and stout rods, somewhat branched	Clusters of rod-shaped spicules
Data source	Bribiesca-Contreras et al. 2022; this study	This study	This study	Koehler and Vaney 1905; Hansen 1975	Ludwig 1893; Hansen1975	Théel 1879; Hansen 1975	Théel 1879; Hansen 1975

‘–’means data not available.

#### ﻿ Oneirophanta lucerna sp. nov.

Taxon classificationAnimaliaSynallactidaDeimatidae

F41B2E8F-675E-5DAF-8DA3-3FBCCCCD95B2

https://zoobank.org/DF0BA6C4-E07A-467A-9983-603B303CDA6E

Figs 6 , 7

##### Type material.

***Holotype*.**IDSSE-2023-0208-HS02, northern slope of the South China Sea, station SY530-HS02 (16°28.60'N, 110°18'E), depth 1389 m, 8 Feb. 2023, preserved in absolute alcohol. ***Paratypes*.** Three specimens. IDSSE-2023-0204-HS02, northern slope of the South China Sea, station SY529-HS02 (16°28.20'N, 110°43'E), depth 1393 m, 4 Feb. 2023, preserved in absolute alcohol. IDSSE-2023-0208-HS01, northern slope of the South China Sea, station SY530-HS01 (16°28.30'N, 110°43'E), depth 1389 m, 8 Feb. 2023, preserved in absolute alcohol. IDSSE-2023-0208-HS03, northern slope of the South China Sea, station SY530-HS03 (16°28.18'N, 110°43'E), depth 1392 m, 8 Feb. 2023, preserved in absolute alcohol.

##### Type locality.

Northern slope of the South China Sea, depth 1389 m.

##### Diagnosis.

Body elongated, skin smooth, color orange in vivo. Mouth anteroventral, anus posteroventral. Tentacle 19 or 20. Ventrolateral tube feet 11–14 on each body side, arranged in single rows. Midventral tube feet two and rudimentary. Dorsal papillae 15–27 on each side, placed in single rows along dorsal radius. Ventrolateral papillae 7–10 on each side, arranged in single rows. Dorsal deposits spatulated crosses, spatulated rods, and widely scattered spinous rods with branched spines. Papillae deposits with spinous rods and spatulated rods. Spatulated crosses with the arms twice divided and two types of spinous rods are in ventrum. Spinous rods and sturdy spatulated rods with open ramifications in tentacles and tube feet.

##### Description of holotype.

**Figure 6.**
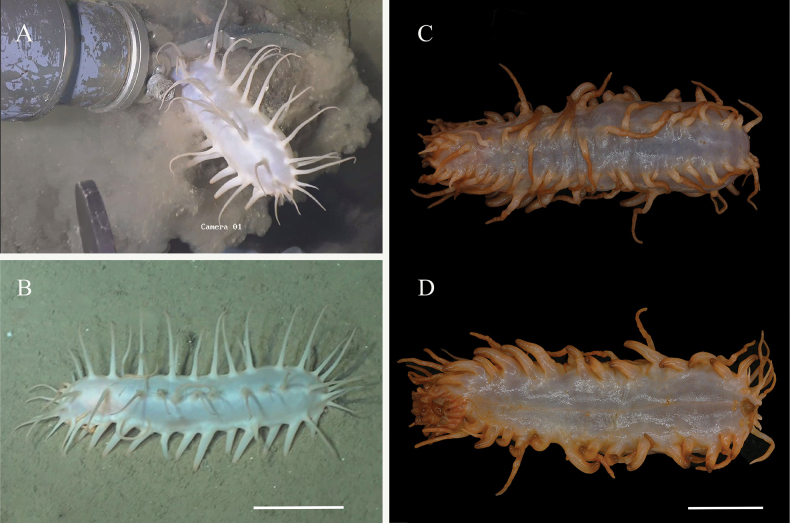
*Oneirophantalucerna* sp. nov. (Holotype IDSSE-2023-0208-HS02) **A, B** in situ images **C** dorsal view **D** ventral view. Scale bars: 5 cm.

***External morphology*.** Body elongated, dorsum inflated, ventrum flattened, slightly narrowed anteriorly (Fig. 6). Length in vivo 22 cm (Fig. 6B), in ethanol 20 cm. Maximum body width 6 cm in vivo, 5.7 cm in ethanol. Color in vivo orange (Fig. 6C), tentacle and tips of papillae and tube feet more pigmented than the body skin. Tentacle 20. Circum-oral papillae absent. Mouth anteroventral, anus posteroventral terminal. Ventrolateral tube feet 14 pairs, placed in single rows on ventrolateral ambulacrum, partly projecting horizontally from the body (Fig. 6D). Midventral radius naked. Midventral tube feet two and rudimentary, with one placed on half the body, while the other placed on a rear quarter of the body, and several smaller tube feet close to anus. Left dorsal papillae 27, right dorsal papillae 21, measuring 1.5–5.2 cm, arranged in regular single rows along dorsal radii (Fig. 6C). Ventrolateral papillae 9–10 on each side, measuring 2.2–3.2 cm, placed in single rows along the ventrolateral radii.

**Figure 7.**
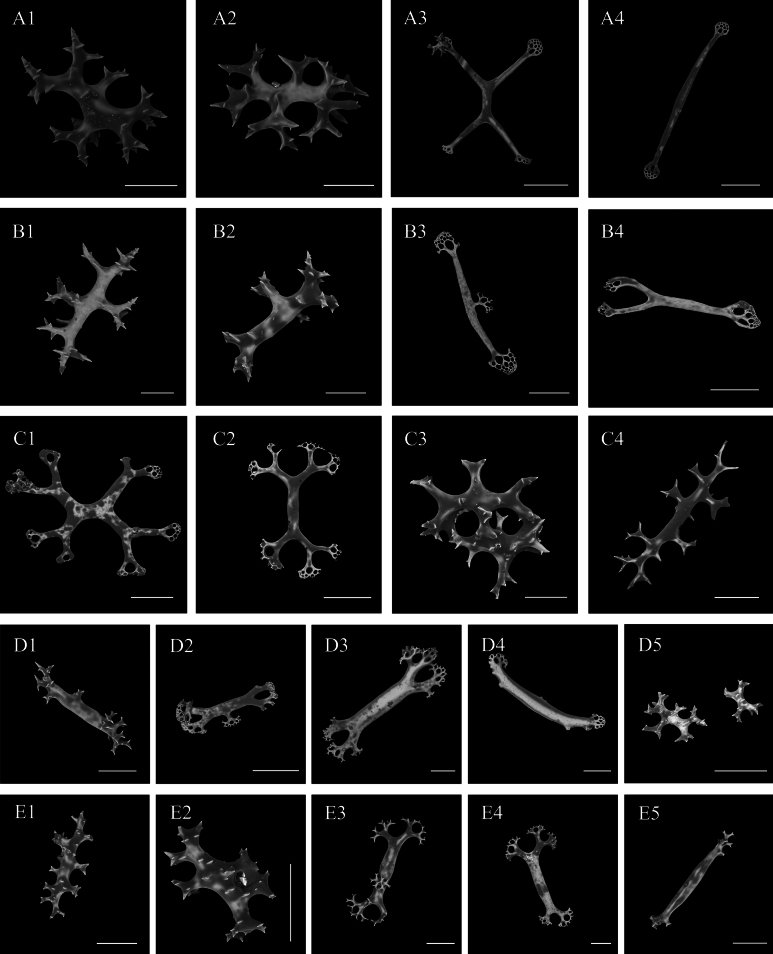
SEM images of *Oneirophantalucerna* sp. nov. (Holotype IDSSE-2023-0208-HS02) **A1–A4** dorsal body wall **B1–B4** papillae **C1–C4** ventral body wall **D1–D5** tube feet **E1–E5** tentacles Scale bars: 50 μm (**A1–A2**, **B1–B2**, **C2**); 100 μm (**C3**, **D1–E5**); 200 μm (**A3**, **A4**; **B3–C1**).

***Ossicle morphology*.** Dorsal deposits contain spatulated crosses, spinous rods and spatulated rods. Spinous rods with irregular spines, 0.1–0.2 mm in length (Fig. 7A1, A2); spatulated crosses with arms 0.2–0.8 mm in length (Fig. 7A3); spatulated rods up to 1 mm (Fig. 7A4). Papillae with spinous rods 0.2–0.3 mm in length (Fig. 7B1, B2), and spatulated rods 0.4–0.9 mm in length, some with an extra branch from central part (Fig. 7B3), some bifurcated at the end (Fig. 7B4). Ventral body wall spatulated crosses with the arms twice divided (Fig. 7C1, C2) and the spinous rods of two types, one with more complex and irregularly placed spines (Fig. 7C3), the other with fewer and more regularly arranged spines (Fig. 7C4), up to 0.4 mm long. Tentacles and tube feet with similar ossicle types, spinous rods of two types, a few regularly distributed spines, 0.3–0.35 mm in length (Fig. 7D1, E1), irregularly shaped (Fig. 7D5, E2), and somewhat sturdy spatulated rods with perforated extremities that occasionally bifurcated (Fig. 7D2–D4, E3, E4), 0.2–0.7 mm in length.

##### Etymology.

The species was named after the Latin word *lucerna* to commemorate the traditional Chinese Lantern Festival, which was relatively close to the time these specimens were collected.

##### Distribution.

Northern slope of the South China Sea, depths of 1389–1393 m.

##### Remarks.

The new species conformed to the genus *Oneirophanta* characterized by uncontracted the tentacles, the absence of oral papillae and tentacle discs without ramified processes. *Oneirophantalucerna* sp. nov. differed from other species of *Oneirophanta* in possessing highly variable shaped spinous rods and ventrolateral tube feet that are only arranged in single rows, whereas, in other species (Table 2), they are arranged in two or three rows along ventrolateral ambulacrum: *O.conservata*, *O.setigera*, and *O.mutabilis* in double rows, *O.idsseica* sp. nov. in alternating two or three rows, and *O.brunneannulata* sp. nov. in three rows. The three accepted species of *Oneirophanta* and the other two new species in this study all have perforated plates that are absent in *Oneirophantalucerna* sp. nov. This is the first record of a species in the genus *Oneirophanta* with mainly irregular spinous rods.

#### ﻿ Oneirophanta mutabilis mutabilis

Taxon classificationAnimaliaSynallactidaDeimatidae

Théel, 1879

A37ABE19-7D9A-5F12-948A-6B5857E14161

Figs 8 , 9

Oneirophanta mutabilis Théel, 1879: 6–7, figs 4–6; Théel 1882: 62–68, pls XXI: 2, XXII, XXXI: 1–3, XXXVI: 1, 2, 8–11, XXXVII: 4, 13, XXXVIII: 11–12, XL: 1–3, XLI: 1, 2, 4, XLII: 9, XLIII: 1, 6, XLV, XLVI: 6, 7; R. Perrier 1902: 374–380, pl. XVIII: 10–15; Grieg 1921: pl. II: 1, 2; Hérouard 1923: 39–40, pls IV: 10, V: 3, 4; Agatep 1967: 63–65, pl. X: 1–7.
Oneirophanta mutabilis mutabilis Théel: Hansen 1967: 485–488, figs 3, 4; Hansen 1975: 24–32, figs 2–5.
Oneirophanta alternata R. Perrier, 1900: 117–118; R. Perrier 1902: 380–386, pls XIV: 3, 4, XVIII: 16–22.
Oneirophanta alternata var. talismani R. Perrier, 1902: 386–388, fig. 6.

##### Material examined.

One specimen. IDSSE-2020-1203-HS01, in the Mariana fore-arc area, western Pacific Ocean, station SY310-HS01 (11°41.42'N, 140°58.56'E), depth 3394 m, 3 Dec. 2020, preserved in absolute alcohol.

##### Description.

**Figure 8.**
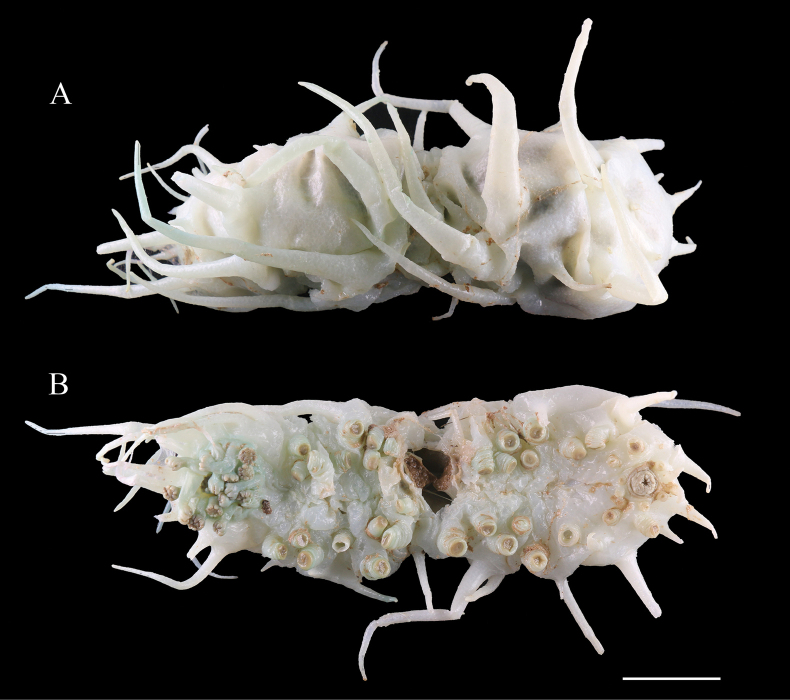
*Oneirophantamutabilismutabilis* Théel, 1879 (IDSSE-2020-1203-HS01) **A** dorsal view **B** ventral view. Scale bar: 3 cm.

***External morphology*.** Body cylindrical, nearly equal in width throughout the whole length and tapering anteriorly. 15 cm long and 4.5 cm wide after fixation with 95% alcohol for several days (Fig. 8A, B). Skin white, calcified and brittle. Tentacles 20, unretractile, 0.6–1.6 cm in length, with 7–9 marginal digits. Mouth and anus ventral. Ventrolateral ambulacra with 16 tube feet on each side, arranged in two irregular rows. Dorsal papillae 6 pairs, the maximum length up to 10 cm, placed in single rows along dorsal radii. Ventrolateral papillae 13 on each side, the maximum length up to 6 cm. Midventral tube feet not found due to damage to the mid-abdomen of the specimen, and only approximately six small tube feet observed near the anus.

**Figure 9.**
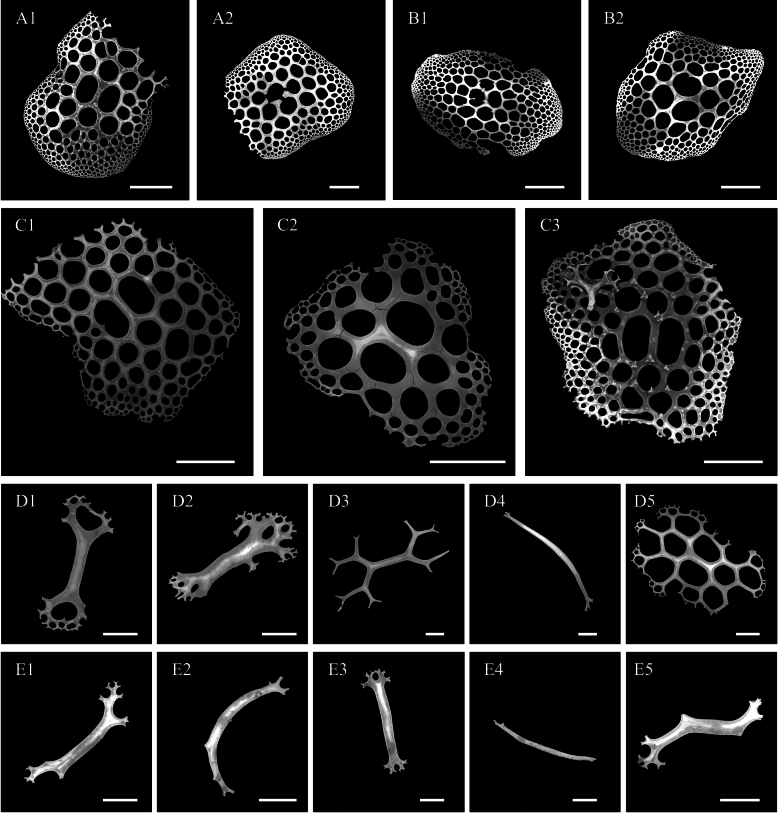
SEM images of *Oneirophantamutabilismutabilis* Théel, 1879 (IDSSE-20201203-HS01). **A1, A2** dorsal body wall **B1, B2** dorsal papillae **C1–C3** ventral body wall **D1–D5** tube feet **E1–E5** tentacle. Scale bars: 300 μm (**A1–C3**); 100 μm (**D1–E1**, **E3–E5**); 50 μm (**E2**).

***Ossicle morphology*.** The body wall and papillae with perforated plates (Fig. 9A1–C3), 0.9–1.5 mm in diameter. The entire periphery of fully developed plates usually surrounded by closed holes, with large central holes (especially in ventrum) and small peripheral holes. Some plates bearing 8–14 small, vertical spines (Fig. 9B1, C3), but no formation of a secondary layer of meshwork. Tube feet with rods up to 0.8 mm long (Fig. 9D1, D2, D4) and incompletely developed plates with open ramifications (Fig. 9D3, D5). The irregular rods in tentacles with variable bending angles (Fig. 9E1–E5).

##### Distribution.

Cosmopolitan, depth 2515–6000 m (Hansen 1975, 1967; Thandar 1984).

##### Remarks.

*Oneirophantamutabilis* was first described west of the Crozet Islands (H.M.S. Challenger station 146: 46°46'S, 45°31'E) at depths of 2514 m (Théel 1879). It was divided into two subspecies, *Oneirophantamutabilismutabilis* Théel, 1879 and *Oneirophantamutabilisaffinis* Ludwig, 1893, based on differences in tentacle shape, the type of deposit, and the number of dorsal papillae (Hansen 1967). There are four main distinctions between them (see Table 2): (1) *O.m.affinis* was restricted to a relatively small area in the eastern Pacific where it replaced the otherwise cosmopolitan *O.m.mutabilis*. (2) tentacles with marginal digits were found in all specimens examined of *O.m.mutabilis*, but tentacle discs of *O.m.affinis* were smooth or had incised edges, and they lacked marginal digits. (3) the perforated plates of *O.m.affinis* were almost completely devoid of vertical spines, whereas those of *O.m.mutabilis* had vertical spines. (4) numerous rods of *O.m.affinis* were usually present in the tentacle discs, which was an additional difference from *O.m.mutabilis*. The morphological characteristics of our specimens were in accordance with the description of *O.m.mutabilis* (Hansen 1975). This was the first record of *O.m.mutabilis* from the Mariana fore-arc area.

**Genus *Deima* Théel, 1879**

#### ﻿ Deima validum validum

Taxon classificationAnimaliaSynallactidaDeimatidae

Théel, 1879

F0EE0E3B-D5DA-5A21-A0A1-6945C9683DD6

Figs 10 , 11

Deima validum Théel, 1879: 5, figs 36–38; Théel 1882: 68–70, pls 18, 19, 31: 4–9, 36: 4, 37: 8, 43: 7, 44: 13, 46: 5; Sluiter 1901a: 60.
Deima validum validum : Hansen 1967: 488–490, fig. 5; Hansen 1975: 17–23, fig. 1, pls 11 (fig. 1), 13, (figs 1, 2); Bohn 2006: 9, fig. 4; Fernández-Rodríguez et al. 2019: 298, fig. 6.
Deima fastosum Théel, 1879: 5–6, figs 1–3; Théel 1882: 71–73, pls 20, 21: 1, 31: 10–13, 35: 7–10, 36: 7. 37: 3, 43: 2–3, 5, 46:8.
Deima blakei Théel, 1886b: 1–2, figs 1, 2; Koehler and Vaney 1905: 55–57, pl. 11: 13–15; Hérouard 1923: 40–41, pls. 5: 7, 6: 5; Deichmann 1930: 115–116, pls 10: 7–11, 11: 1–3; Deichmann 1940: 198–199.
Deima atlanticum Hérouard, 1898: 88–89, figs 1, 2.
Deima mosaicum Ohshima, 1915: 233–234.

##### Material examined.

Two specimens. IDSSE-2019-0630-HS01, collected from the northern slope of the South China Sea, station SY155-HS01(17°43'N, 114°13'E), depth 3451 m, 30 Jun. 2019, preserved in absolute alcohol. IDSSE-2018-0531-HS02, collected from the Xisha Trough of the South China Sea, station SY84-HS02 (18°2'N, 114°5'E), depth 3404 m, 31 May 2018, preserved at -80 °C.

##### Description.

**Figure 10.**
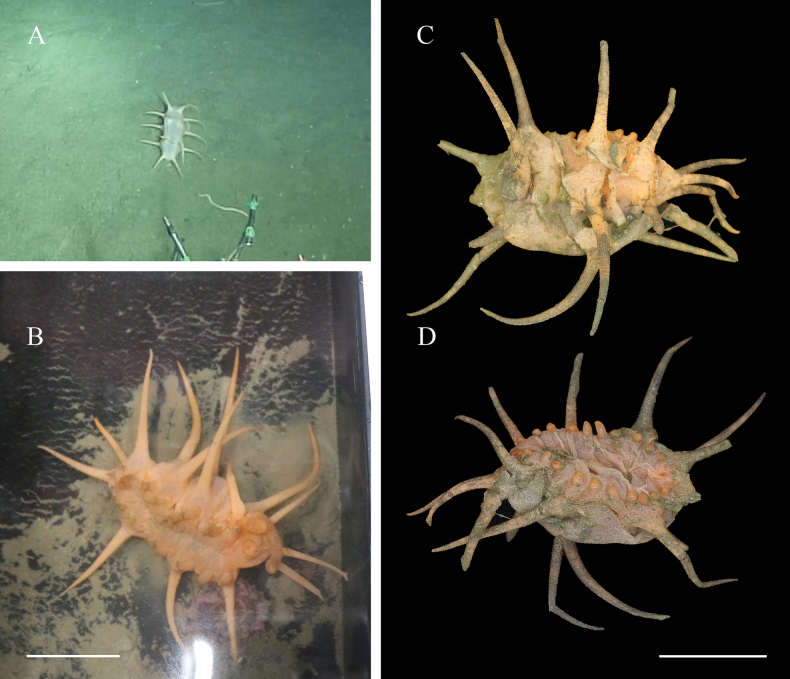
*Deimavalidumvalidum* (IDSSE-2018-0531-HS02 and IDSSE-2019-0630-HS01) **A** in situ image (IDSSE-2018-0531-HS02) **B** in vivo image (IDSSE-2018-0531-HS02) **C** dorsal view (IDSSE-2019-0630-HS01) **D** ventral view (IDSSE-2019-0630-HS01). Scale bars: 5 cm.

***External morphology*.** Body ovate, dorsal vaulted, ventral flattened. 9–10 cm long and average 5.5 cm wide in vivo. Skin rigid, body wall brittle and easily broken. Color orange in vivo (Fig. 10A, B). Tentacles retracted into the mouth, resulting in uncountable quantities (Fig. 10C, D). 12 pairs of ventrolateral tube feet contractible based on morphology of different stages, single-rowed. Five pairs of rigid and conical dorsal papillae placed in two rows. Ventrolateral papillae three pairs, half the length of the body. Pre-anal tube feet absent.

**Figure 11.**
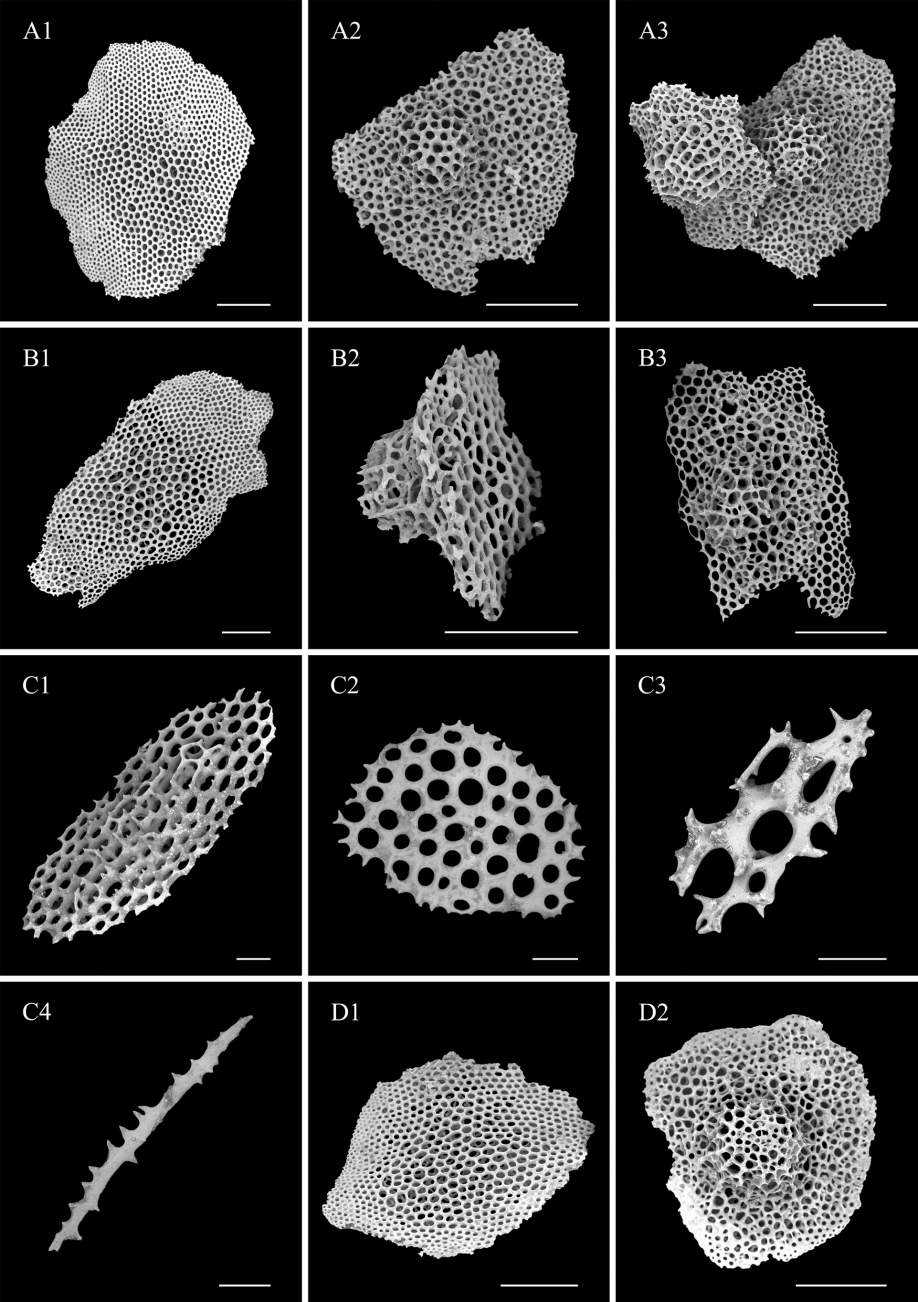
SEM images of *Deimavalidumvalidum* (IDSSE-2019-0630-HS01). **A1–A3** dorsal body wall **B1–B3** papillae **C1–C4** tube feet **D1–D2** ventral body wall. Scale bars: 300 μm (**A1–B3**); 50 μm (**D1**, **D2**)

***Ossicle morphology*.** Basal layer and several additional layers amount in the center of the perforated plates on the body wall and dorsal papillae, in diameter 0.6–1.8 mm (Fig. 11A1–B3, D1, D2), with regular holes and a large, reticulated knob, ~ 0.2 mm high. The tube feet with some types of developmental stages towards perforated plates (Fig. 11C1, C3) and perforated plates with one layer (Fig. 11C2), rods bearing spines (Fig. 11C4).

##### Distribution.

This subspecies probably has a cosmopolitan distribution, except for the Arctic and Southern Ocean, at depths of 724–5426 m (Théel 1882, 1886b; Sluiter 1901a; Hérouard 1902, 1923; Koehler and Vaney 1905; Ohshima 1915; Grieg 1921; Deichmann 1940; Hansen 1975; Sibuet 1977; O’Loughlin 1998; Bohn 2006).

##### Remarks.

*Deimavalidum* was first described by Théel (1879) in the preliminary report of the exploring voyage of H. M. S. Challenger under Sir C. Wyville Thomson., with a detailed description of another novel species, *Deimafastosum*. Four new species were reported subsequently by different taxonomists: *D.atlanticum* Hérouard, 1898; *D.blakei* Théel, 1886; *D.mosaicum* Ohshima, 1915 and *D.pacificum* Ludwig, 1894. The family Deimatidae underwent a thorough revision by Hansen (1975), who regarded *D.fastosum*, *D.atlanticum*, *D.blakei*, *D.mosaicum* and *D.pacificum* as junior synonyms of *D.validum*. Hansen (1967) separated the species into two subspecies, cosmopolitan *Deimavalidumvalidum* and the eastern Pacific Ocean *Deimavalidumpacificum* Ludwig, 1894, based on differences in the number of dorsal papillae and the type of deposit in which they were found.

The two specimens examined here are consistent with the diagnosis of subspecies *D.v.validum* as described in detail by Hansen (1975). Perforated plates were like those of *D.fastosum*, with a large, reticulated, conical knob, which hardly ever approached in other known specimens that were used to investigate *D.v.validum*. This unique feature represents an extreme case of plate variation, where the spinous appearance of the skin is due to these very high and often vertically rising knobs on the plates. Hansen (1975) hypothesized that additional mesh structures on perforated plates varied with depth into two types: one-layered plates were characteristic of bathyal specimens, and many-layered plates were typical of abyssal specimens. However, it also exhibited a transitional type of plate in the abyssal Tasman Sea specimens and the bathyal Japanese specimens (perforated plates that were completely or almost completely devoid of additional meshwork), which prevented a clear distinction between a bathyal and an abyssal type of deposit.

Only in the Bay of Bengal (depth 1224–3365 m) did the development of additional layers of meshwork increase progressively with depth. In this research, high-knobbed plates were present in the abyssal South China Sea specimens (depth > 3000 m), but they were absent from the South China Sea specimens first reported (depth 1100 m) by Liao (1997). This was the second time that *Deimavalidum* has been recorded in the South China Sea. The proposition that the development of additional meshwork on perforated plates increased with depths needs to be investigated on more specimens at different depths in the South China Sea.

### ﻿Genetic distance and phylogenetic analyses

The inter- and intraspecific genetic divergences of the COI gene were calculated to calculate the genetic distances in Deimatidae (Suppl. material 1). For the COI alignment, the interspecific distances in *Oneirophanta* ranged from 8.2–15.3%, the intraspecific distances in *Oneirophanta* were in the range of 0–0.6%, and the range of genetic distances among three genera was 15.1–21.9%.

**Figure 12.**
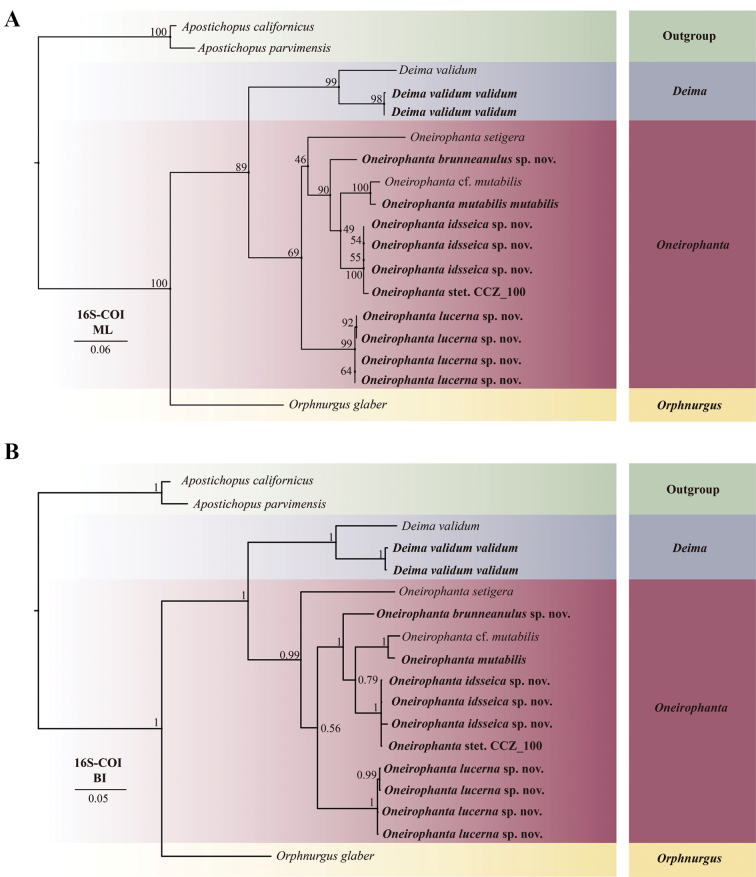
Maximum likelihood (ML) and Bayesian inference (BI) trees based on concatenated 16S-COI sequences showing phylogenetic relationships among deimatid species. The new sequences provided in this study are in bold **A**ML tree, with bootstrap replications labeled **B**BI tree, with posterior probability labeled.

In total, 11 COI sequences and 10 16S sequences were deposited into GenBank (Table 1). To check the intrageneric relationships of species in Deimatidae, a Bayesian phylogenetic tree and a Maximum likelihood (ML) tree were reconstructed using concatenated 16S-COI sequences of length 1191 bp. The result of the phylogenetic analyses showed quite similar topologies in BI and ML trees (Fig. 12), except for one area of the BI tree, where *Oneirophantasetigera* formed an independent clade within *Oneirophanta* (BI 0.99).

The phylogenetic relationships of Deimatidae clustered into three portions and were consistent with the traditional classification system (Fig. 12). Portion 1: *Deimavalidum* was clustered with *Deimavalidumvalidum* (ML 99%, BI 1), which formed a monophyletic sister group (ML 89%, BI 1) with *Oneirophanta*. Portion 2: The three new species and the new record fell into *Oneirophanta*, which was divided into three clades. Clade 1: *O.setigera* was shown to be distinct from the other congeners in the BI tree (BI 0.99). But in the ML tree, *O.setigera* clustered with *O.brunneannulata* sp. nov., O.cf.mutabilis, *O.mutabilis* and *O.idsseica* sp. nov. with low support (ML 46%). Clade 2: *O.idsseica* sp. nov. clustered with O.cf.mutabilis and *O.mutabilis* (ML 49%, BI 0.79), followed by *O.brunneannulata* sp. nov. (ML 90%, BI 1). Clade 3: *O.lucerna* sp. nov. clustered with all other congeners in the ML tree (ML 69%). But in the BI tree, it was a sister taxon to species in clade 2, which then clustered with *O.setigera*. Portion 3: *Orphnurgusglaber* formed a separate clade with full node support (ML 100%, BI 1), and it was the only species in *Orphnurgus*.

## ﻿Discussion

### ﻿Species delineation and generic assignment

Both the morphology and molecular phylogenetic analyses supported the assignment of the three new species to the genus *Oneirophanta*. The external morphological characteristics in *Oneirophanta* species were quite similar to those in *Orphnurgus*, but *Oneirophanta* never has tentacle discs with ramified processes, and they usually have rounded knobs on the margin. The three new species described in this study conformed to this feature.

*Oneirophantabrunneannulata* sp. nov., *Oneirophantaidsseica* sp. nov. and *Oneirophantalucerna* sp. nov. can be separated from other congeners by ossicle types, the arrangement and the number of dorsal papillae and tube feet. The separations were confirmed by the p-distance analyses, which showed that the uncorrected p-distance for the COI among *O.brunneannulata* sp. nov. and other congeners was 8.2–13.1%; among *O.idsseica* sp. nov. and other congeners was 8.2–14.1%, and among *O.lucerna* sp. nov. and other congeners was 12.1–14.8%. These divergences were much higher than the known intraspecific variation in *Oneirophanta* spp. (0–0.6%) (Suppl. material 1) and, thus, this warranted separation of *O.brunneannulata* sp. nov., *O.idsseica* sp. nov., and *O.lucerna* sp. nov. from other congeners.

The phylogenetic trees (Fig. 12) showed that *O.idsseica* sp. nov. clustered together with *Oneirophanta* stet. CCZ_100 from Clarion-Clipperton Zone, which was deposited in the Natural History Museum, London (voucher number: CCZ_100). Generally, taxonomic units with sequence differences of < 2% are likely to be the same species, and differences > 5% were confidently used to separate different species (Ward et al. 2008). Because the COI p-distance between the two species was 0.6% (Suppl. material 1), the divergences fell within the range of general intraspecific variation; both morphological characters and molecular data suggested that *O.idsseica* sp. nov. and *O.* stet. CCZ_100 are the same species.

### ﻿Geographic distribution of deimatid species

There are a total of three genera and 16 species in the family Deimatidae, which include the three species that we described here. To date, 11 species of Deimatidae have been discovered in the deep water of the Pacific Ocean. *Deima* only includes one species: *Deimavalidum*, which occurs worldwide at depths of 724–5426 m. Nine species of *Orphnurgus* are accepted, with five species recorded from the Pacific: *Orphnurgusdorisae* Pawson, 2002 from the southern Pacific Ocean (New Zealand), *Orphnurgusglaber* Walsh, 1891 from the central and western Pacific Ocean, *Orphnurgusprotectus* (Sluiter 1901) and *Orphnurgusbacillus* Cherbonnier & Féral, 1981 from the western Pacific Ocean (Celebes Strait and Philippines), and *Orphnurgusvitreus* (Fisher 1907) from the North Pacific Ocean (off Hawaiian Islands). On a vertical scale, all species in this genus inhabited the water from relatively shallow depths to the bathyal zone (depth 174–1301 m). Among the six species, which included the three new species of *Oneirophanta*, *Oneirophantamutabilis*, the type species of the genus, is a cosmopolitan species (Hansen 1975). *O.conservata* was the only species recorded from the Indian Ocean (Koehler and Vaney 1905). All the other species were found mainly in the Pacific Ocean: *O.setigera* from the southern and eastern Pacific (Kermadec Trench and Gulf of Panama), *O.mutabilismutabilis* is a cosmopolitan species with multiple records in the Pacific Ocean (Hansen 1975), and *O.mutabilisaffinis* is restricted to a relatively small area in the eastern Pacific Ocean. The three new species were from the western Pacific Ocean (South China Sea). In addition, *O.conservata* has the shallowest record of the genus (depth 1315 m), and *O.mutabilis* has the deepest record (depth 6000 m). *Oneirophanta* was distinguished from the other two genera by its inhabitance in the bathyal-abyssal zone.

Based on their distribution, deimatid holothurians are abundant in the Pacific Ocean and inhabit a wide range of depths (174–6000 m). Future expeditions to the Pacific zone may discover even more species, and more research is needed to evaluate the species diversity and geographic distribution of these deep-sea holothurians.

## Supplementary Material

XML Treatment for Oneirophanta idsseica

XML Treatment for Oneirophanta brunneannulata

XML Treatment for Oneirophanta lucerna

XML Treatment for Oneirophanta mutabilis mutabilis

XML Treatment for Deima validum validum

## ﻿Additional information

### Author ORCIDs

Haibin Zhang https://orcid.org/0000-0001-5429-9851

### Data availability

All of the data that support the findings of this study are available in the main text or Supplementary Information.

## Supplementary materials

Supplementary material 1 Estimates of p-distances of the COI gene among deimatid species and studied sequences Data type docx https://binary.pensoft.net/file/1006164This dataset is made available under the Open Database License (http://opendatacommons.org/licenses/odbl/1.0/). The Open Database License (ODbL) is a license agreement intended to allow users to freely share, modify, and use this Dataset while maintaining this same freedom for others, provided that the original source and author(s) are credited.Yunlu Xiao, Haibin Zhang
