# Supplementary material for: ﻿Three new species and one new record of Deimatidae (Echinodermata, Holothuroidea, Synallactida) discovered in the South China Sea and the Mariana fore-arc area using integrative taxonomic methods
Source: Zookeys. 2024 Mar 20;1195:309–35. doi: 10.3897/zookeys.1195.115913 (PMC10973770; doi:10.3897/zookeys.1195.115913)
Supplement: Supplementary material 1 — Estimates of p-distances of the COI gene among deimatid species and studied sequences [file zookeys-1195-309_article-115913__-s001.docx]

**Table S1.** Estimates of p-distances of the COI gene among deimatid species and studied sequences.

|  | **1** | **2** | **3** | **4** | **5** | **6** | **7** | **8** | **9** |
| --- | --- | --- | --- | --- | --- | --- | --- | --- | --- |
| 1 *Oneirophanta brunneannulata* sp. nov. | – |  |  |  |  |  |  |  |  |
| 2 *Oneirophanta lucerna* sp. nov. | 12.1–12.5% | **0–0.3%** |  |  |  |  |  |  |  |
| 3 *Oneirophanta* stet. CCZ–100 | 9.4% | 14.5–14.8% | – |  |  |  |  |  |  |
| 4 *Oneirophanta idsseica* sp. nov. | 8.2% | 13.0–13.6% | **0.6%** | **0** |  |  |  |  |  |
| 5 *Oneirophanta mutabilis* | 9.3% | 13.8–14.3% | 10.1% | 8.4–8.5% | – |  |  |  |  |
| 6 *Oneirophanta* cf. *mutabilis* | 11.3% | 13.9–14.3% | 10.0% | 10.1–10.3% | 2.2% | – |  |  |  |
| 7 *Oneirophanta setigera* | 13.1% | 13.6–14.0% | 15.3% | 14.0–14.1% | 12.2% | 12.9% | – |  |  |
| 8 *Deima validum* | 15.4–15.6% | 15.1–15.6% | 20.1–21.2% | 18.6–19.3% | 17.1–17.3% | 18.9–20% | 16.8–17.0% | **0.5%** |  |
| 9 *Orphnurgus glaber* | 19.6% | 17.9–18.5% | 20.1% | 19.3–19.4% | 19.3% | 21.9% | 20.9% | 19.4–19.5% | – |

Intraspecific distances are in bold. ‘–’ means no data.
